# Parental sociodemographics of medically assisted reproduction births in the United States: a dyadic population-level study

**DOI:** 10.1016/j.xfre.2023.05.008

**Published:** 2023-06-01

**Authors:** Ester Lazzari, Katherine Tierney

**Affiliations:** a Department of Demography, University of Vienna (Wittgenstein Centre for Demography and Global Human Capital (IIASA, OeAW, University of Vienna)), Vienna, Austria; b Department of Sociology, Western Michigan University, Kalamazoo, Michigan

**Keywords:** Reproductive technologies, population-based studies, parental characteristics, social disparities, United States

## Abstract

**Objective:**

To study how men’s and couples’ sociodemographic characteristics predict the probability of having a birth conceived using medically assisted reproduction (MAR) in the United States.

**Design:**

Population-based study.

**Setting:**

Not applicable.

**Patient(s):**

Men and women in the National Vital Statistics Birth certificate data from 2009 to 2019.

**Intervention:**

None.

**Main Outcome Measure(s):**

Proportion of MAR births out of total births by parental sociodemographic categories and probability of having a MAR birth.

**Result(s):**

Between 2009 and 2019, the overall prevalence of MAR births among men was 1.81%. Fathers of children conceived using MAR tended to be older, higher educated, and white compared with fathers of naturally conceived children. During the period of 2009–2019, these sociodemographic profiles remained largely unchanged. Controlling for maternal age and birth order only partially reduced disparities by education and race. In 2019, highly educated fathers were 2.04 percentage points (95% confidence interval, 1.97–2.12) more likely to have a MAR-conceived birth than fathers with a low educational level, and black fathers were associated with a reduction in the probability of having an MAR-conceived child by −1.07 percentage points (95% confidence interval, −1.11 to −1.04) compared with white fathers. The dyadic analysis using parents’ education and race interactions revealed that partnering with someone of a higher educational level increases the likelihood of having a MAR birth, beyond what would be observed by considering only individual-level characteristics.

**Conclusion(s):**

To comprehend the environment in which MAR–conceived children are born and raised, performing dyadic analyses that examine the characteristics of both partners is essential. The findings underscore the enduring presence of substantial social disparities in MAR use in the United States, with MAR-conceived children raised in environments of relative advantage, which may impact their future health and development.

Since the introduction of medically assisted reproduction (MAR), which comprises fertility treatments such as ovulation induction, intrauterine insemination, and all assisted reproductive technology (ART) procedures, including in vitro fertilization, its utilization has grown (1). For example, in the United States, the number of cycles of in vitro fertilization increased from 64,583 in 1996 to 209,687 in 2019 (2, 3). Similar or greater growth has been observed in other European countries where treatments are more affordable and accessible (4).

Several social and demographic conditions contribute to these trends. Postponement of first births is one such factor, which is, in turn, connected to increases in women’s education and labor force participation. Because fecundity for women and men declines with age, delays to first births are associated with increases in difficulties becoming pregnant and infertility (5, 6), which have led to the higher utilization of ART (5). Although women may recuperate some postponed births (7, 8), there is little evidence that the postponement trend or related social trends are changing (9). Additionally, increasing acceptance of same-sex parents as well as the medicalization of infertility may contribute to the continued and increased use of MAR (10). Recent demographic projections and forecasts in Australia and the United States estimated that the use of ART treatments and share of ART births will likely continue to increase in the future (11, 12, 13), demonstrating the continued importance of MAR births in high-income, low-fertility contexts.

MAR technologies are especially important for women at older ages and for first births (14, 15, 16) and may be the only option for biological children for some people. Hence, understanding utilization patterns and inequalities in these technologies is vital. Previous studies analyzing the sociodemographic characteristics of people using MAR have mainly focused on women, partly because of data limitations and the socially gendered nature of reproduction (17).

However, there has been an increased call for reproduction research to include men (17, 18, 19, 20, 21, 22, 23). Notably, up to half of infertility cases are identified as due to male factors, and a few studies have found an association between paternal age and MAR outcomes (24, 25, 26). In the United States, surveys have shown men who seek infertility care tend to be married, have higher levels of education and incomes, and are more likely to have private insurance than men who do not report treatment seeking (27, 28). The small and growing body of work on the sociodemographic of men with MAR- or ART-conceived children has also shown these men to be older with higher levels of education than those who have naturally conceived children (14, 29, 30, 31).

A dyadic approach that examines the relationship between women and men involved in MAR births may also be valuable. This approach is increasingly common in studies about coping and experiences with infertility (e.g., studies by Benyamini et al. (32), Johnson and Johnson (33), Hammarberg et al. (34), Martins et al. (35), Taylor (36), and Lazzari et al. (37)). Sociodemographic research has begun to explore the importance of parental characteristics jointly, such as the age of women and men presenting for infertility treatment (e.g., the study by Stern et al. (38)), and combining parental socioeconomic status into a single measure for investigation (14, 31, 39). Yet, continued efforts to assess the interrelation between parental characteristics and MAR are still needed to better identify potential social inequities in utilization.

Socioeconomic, racial, and ethnic disparities in access to MAR are especially relevant in the United States, where the out-of-pocket cost for an ART cycle is among the highest in the world and the healthcare system is socially stratified (40). Specifically, several studies have shown that women of color and women with lower levels of education experience higher or equivalent rates of infertility but are underrepresented in the use of most infertility treatments (e.g., studies by Tierney and Cai (16), Fujimoto et al. (41), Greil et al. (42), Chin et al. (43), Peck et al. (44), and Shirazi and Rosinger (45)).

Although research on MAR and men is growing, there is currently no population-level study using US data that analyzes the sociodemographics of men involved in MAR births or the interrelationships between men and women involved in MAR births. Additionally, there is little evidence on how the probability of having a MAR birth varies by the racial and educational pairings of the couple. Thus, our study aimed to fill these gaps by analyzing the sociodemographics of men involved in MAR births and investigating how the interrelationships between women and men involved in MAR births is associated with the MAR birth rates in the United States. This study is an important contribution to the literature because it addresses the underresearched area of paternal and racial/ethnic factors in MAR births.

## Materials and Methods

### Data

This study draws on publicly available birth certificate data sourced from the National Vital Statistics System (NVSS), an intergovernmental data sharing program by which the National Center for Health Statistics collects and disseminates official vital statistics. The certificates report on a variety of parental sociodemographic and health data, including indicators of whether the birth was conceived using MAR. Since reporting of fathers’ sociodemographic characteristics began in 2009, our analysis covers the period of 2009–2019. After excluding births with missing information on maternal socioeconomic variables (n = 4,409,990), paternal sociodemographic variables (n = 5,466,207), and those with unknown mode of conception (n = 56,195), the total sample consisted of 32,847,785 births, of which 594,463 were MAR-conceived. Whenever possible, the NVSS imputed data were used. An institutional review board approval was not obtained because the study does not include any interaction with human subjects.

### Outcome and Dependent Variables

The binary outcome variable indicated whether a live birth was conceived using MAR. The dependent variables collected on birth certificates in the United States were the mother’s and father’s education, the mother’s and father’s race, maternal age at birth, and birth order (on the basis of the number of prior live births to the mother).

At the individual level, education was categorized as follows: less than high school; high school; some college; 4-year degree; and more than 4-year degree. Because few couples feature a combination of the highest and lowest educational categories, in the multivariate analysis, the 2 lowest educational groups were combined. The race variable distinguished men who were white, non-Hispanic (NH); black, NH; Hispanic; and Asian, NH, and the residual category of “Others, NH.”

### Statistical Analyses

First, we showed the results from a simple bivariate analysis describing the proportion of MAR-conceived births out of total births by the age, race, and educational attainment of fathers and mothers between 2009 and 2019. We did not present the detailed descriptive statistics for mothers because these were available elsewhere (24). Subsequently, the linear probability models (LPMs) were estimated to investigate the association between the sociodemographics of parents and having a MAR-conceived birth. We started by analyzing data from 2019 to assess potential social inequities in the most recent year of data in the study. Then, we repeated the analysis for the period of 2009–2019. The LPM used a binary outcome coded as 1 if the birth was MAR-conceived and 0 otherwise. The coefficients indicated the probability that a MAR-conceived birth occurred (46). Such probability was estimated after accounting for the potential confounding factors of maternal age and birth order. Although maternal age had been used in previous studies as a proxy for need (14, 15), the inclusion of birth order was a unique feature of this analysis. We used robust standard errors because the data violated the heteroskedasticity assumption.

The analysis used stepwise procedures to examine the association between paternal and maternal characteristics and the probability of having a MAR-conceived birth. Educational and racial pairings were explored by interacting the father’s and mother’s education and race. Models were adjusted for maternal age and the number of maternal live-born siblings and evaluated for statistical significance and fit using the Bayesian and Akaike information criteria.

### Sensitivity Analyses

We repeated the regression analysis using only the age of the male partner to verify the stability of our findings. Additionally, predictive mean matching was used to estimate missing paternal records for age, race, and education because of underreported paternal information in the NVSS birth certificate data (47). The results from both analyses were consistent with our presented findings.

## Results

### Proportion of MAR Births by Paternal Sociodemographic Characteristics

**Figure 1:**
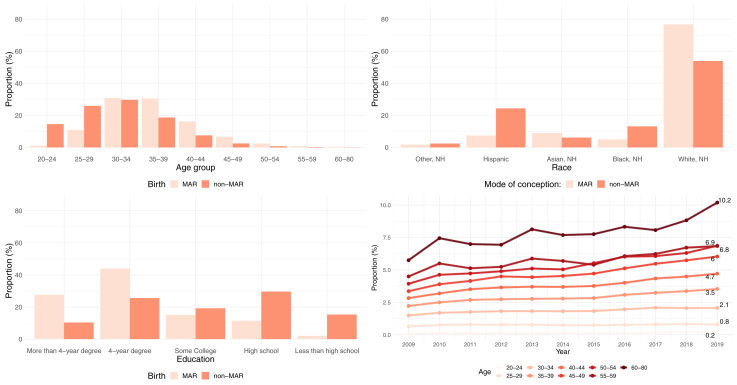
Distribution of medically assisted reproduction (MAR)–conceived and non–MAR-conceived births by paternal sociodemographic characteristics, 2009–2019, United States. Source: Investigators’ calculations on the basis of the National Vital Statistics System. NH = non-Hispanic.

Between 2009 and 2019, the overall prevalence of MAR births among men was 1.81%. Figure 1 shows how the proportion of MAR births varied by paternal sociodemographic characteristics during the 9-year study period. The age distributions of MAR- and non–MAR-conceived births significantly differed as 57.1% of MAR-conceived births occurred among fathers aged ≥35 years compared with only 29.5% of non–MAR-conceived births. Conversely, MAR-conceived births were substantially less prevalent before the age of 30 years: 12.0% of MAR-conceived births were to fathers aged 20–29 years compared with 40.0% of non–MAR-conceived births. White, NH men (76.7%) and those with more than a 4-year degree (27.6%) had a higher prevalence of MAR-conceived births. Over the study period, the prevalence of MAR-conceived births increased at all ages, with fathers aged 45–49 and 50–54 years experiencing the fastest growth, increasing by 76.5% and 77.9%, respectively (Fig. 1).

### Racial and Educational Pairings

**Figure 2:**
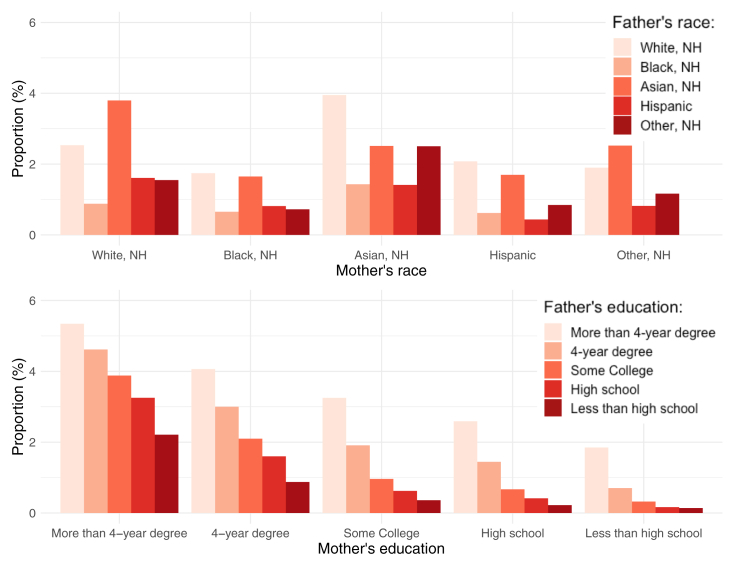
Proportion of medically assisted reproduction out of total births by parental racial and educational pairings, 2009–2019, United States. Source: Investigators’ calculations on the basis of the National Vital Statistics System. NH = non-Hispanic.

Figure 2 displays the prevalence of MAR-conceived births out of total births by racial pairing combinations of parents. White, NH (2.5%) and Asian (2.5%) parents and interracial Asian-white, NH couples had higher prevalence rates (3.9% for white, NH father/Asian mother couples and 3.8% for Asian father/white, NH mother couples). Among couples in which the mother was white, NH, the prevalence of a MAR-conceived birth decreased to 1.6% if the father was Hispanic and 0.9% if the father was black, NH. Among couples in which the father was white, NH, the prevalence of a MAR-conceived birth decreased to 2.1% if the mother was Hispanic and 1.7% if the mother was black, NH. Compared with white, NH or Asian couples, black, NH and Hispanic couples were approximately 4 times and 6 times less likely to have a MAR-conceived birth.

### Multivariate Analyses

**Figure 3:**
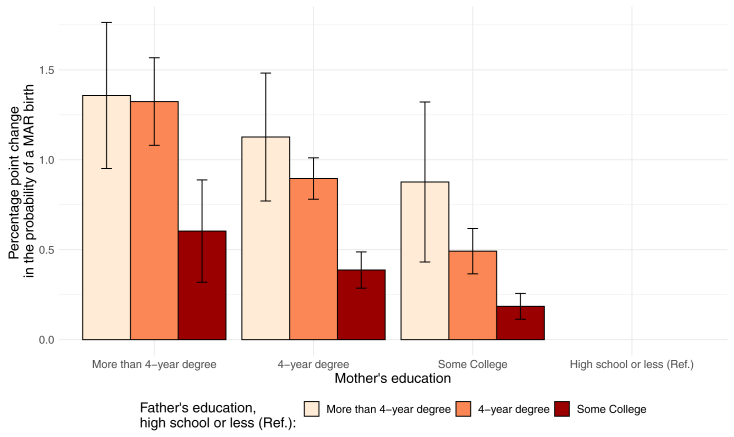
Percentage point change in the probability of a medically assisted reproduction (MAR)–conceived birth (with 95% confidence interval), by parental educational pairings (2019 births). Interaction only for the year 2019. All interactions were statistically significant (*P*< .01). Source: Investigators’ calculations on the basis of the National Vital Statistics System.

The results from the LPM showed differences in the probability of having a MAR-conceived child by paternal race and education, net of confounders (maternal age and birth order). Parallel analyses adjusting for paternal age showed similar results to those presented in Table 1 (not shown). The findings are presented stepwise: model I reports the main effects of paternal race and education, whereas model II adds maternal sociodemographic characteristics. Figure 3 shows the interaction between mothers’ and fathers’ educational attainment (more details shown in Supplemental Table 1, available online) obtained by fitting an additional regression model with an interaction term. The multivariate regression analyses indicated that in 2019, net of the other covariates, white, NH, fathers were more likely to have a MAR-conceived birth than fathers from any other racial group (Table 1). For example, compared with white, NH fathers, black, NH fathers were associated with a reduction in the probability of having a MAR-conceived child of −1.07 (95% confidence interval [CI], −1.11 to −1.04), net of all other covariates. This was the largest difference observed on the basis of race. Contrary to what was observed in the bivariate analyses, Asian, NH fathers had a lower probability of having a MAR-conceived child than white, NH fathers, net of other covariates. Statistically significant differences in the probability of a MAR-conceived birth were also observed on the basis of educational attainment. Indeed, fathers with more than a 4-year degree were 2.04 percentage points (95% CI, 1.97–2.12) more likely to have a MAR-conceived birth than fathers with less than a high school degree, net of all other covariates. The addition of maternal characteristics in model II had a moderating effect on the coefficients, revealing similar racial and educational patterns among mothers (Table 1).

**Table 1:** Linear probability models for medically assisted reproduction–conceived births (2019 births)

Parameter	Model 1, paternal sociodemographics		Model 2, paternal and maternal sociodemographics	
	Coefficient	95% CI	Coefficient	95% CI
Father’s race				
White, NH (Ref.)	0.00		0.00	
Black, NH	−1.07	(−1.11 to −1.04)	−0.72	(−0.79 to −0.66)
Hispanic	−1.03	(−1.06 to 1.00)	−0.62	(−0.67 to −0.56)
Asian, NH	−0.68	(−0.76 to −0.59)	−0.37	(−0.52 to −0.21)
Other, NH	−0.62	(−0.69 to −0.55)	−0.47	(−0.55 to −0.38)
Mother’s race				
White, NH (Ref.)			0.00	
Black, NH			−0.45	(−0.51 to −0.38)
Hispanic			−0.55	(−0.60 to −0.50)
Asian, NH			−0.13	(−0.57 to −0.27)
Other, NH			0.00	(−0.21 to −0.05)
Father's education				
More than 4-y degree	2.04	(1.97–2.12)	1.65	(1.56–1.73)
4-y degree	0.70	(0.66–0.74)	0.62	(0.57–0.66)
Some college	0.04	(0.00–0.07)	0.08	(0.05–0.12)
HS or less (Ref.)	0.00		0.00	
Mother's education				
More than 4-y degree			0.90	(0.82–0.98)
4-y degree			−0.19	(−0.23 to 0.15)
Some college			−0.25	(−0.28 to −0.22)
HS or less (Ref.)			0.00	
Maternal age	0.37	(0.37–0.38)	0.36	(0.36–0.37)
Birth order				
1 (Ref.)				
2	−1.45	(−1.49 to 1.41)	−1.43	(−1.47 to −1.38)
3	−2.88	(−2.93 to −2.83)	−2.82	(−2.87 to −2.77)
4 and above	−3.65	(−3.70 to −3.59)	−3.56	(−3.61 to −3.50)
N	3,214,645		3,214,645	

*Note:* The coefficients showed the percentage point changes in the probability of the birth being conceived using medically assisted reproduction. The results were obtained by fitting linear probability models. Source: Investigators’ calculations on the basis of the National Vital Statistics System.

CI = confidence interval; NH = non-Hispanic; Ref. = reference.

Further analyses performed over the entire study period displayed comparable sociodemographic profiles associated with a birth resulting from MAR (Supplemental Table 2, available online). We found statistically significant differences in the probability that a birth was MAR-conceived across periods. After controlling for other covariates, births in 2019 were 0.44 percentage points (95% CI, 0.41–0.46) more likely to be MAR-conceived than those in 2009. The percentage point increases in the probability of a birth being MAR-conceived for 2018, 2017, and 2016 were 0.38, 0.36, and 0.26, respectively. These were significantly higher than those at the beginning of the study period, where the coefficients for the years 2010–2015 ranged from 0.12 to 0.16, suggesting that the prevalence of MAR births has been increasing at a faster pace in recent years.

The results of the multivariate regression analysis with interaction terms revealed notable trends. First, when examining the educational attainment of both mothers and fathers, we observed that socioeconomic disparities in the likelihood of having a child conceived through MAR were exacerbated by the educational attainment of the partner. Specifically, the positive impact of the mother’s education on the probability of having a MAR-conceived child became more pronounced as the father’s education improved (as shown in Fig. 3 and Supplemental Table 1). On the other hand, the interaction between races did not exhibit a discernible pattern, and the combined effect of the father’s and the mother’s race was not significantly greater than the sum of their individual effects (Supplemental Table 2). Although some individual terms are significant, they did not translate into a meaningful pattern when examining the broader picture.

The goodness-of-fit tests (Akaike and Bayesian information criteria) revealed that the model using race and educational interactions in addition to the race and educational attainment of the mother and father improved model fit (Supplemental Table 1). This indicated the importance of considering parents’ race and education in tandem when evaluating how these variables impact the probability of having a MAR-conceived child.

## Discussion

Between the years 2009 and 2019, the overall prevalence of MAR births among men was 1.81%. Fathers of children conceived using MAR tended to be older, higher educated, and of white, NH race compared with fathers of naturally conceived children. During the study period, these sociodemographic profiles remained largely unchanged. Our findings align with those of previous studies that documented ethnic and educational disparities in infertility treatment usage among women in the United States (16, 42, 48).

The disparities observed in the use of MAR across the education and race groups are partly because of differences in the timing of childbearing. On average, the age at first birth is increasing in the United States; however, it is approximately 3–5 years higher for women with a college or postgraduate degree than for women with a high school degree or lower educational level. Among men, the mean age at first birth is 6–8 years higher for those with a bachelor degree or more than for those with less than a high school degree (49, 50). There are also disparities in childbearing timing on the basis of race, with black and Hispanic women tending to form larger families and have children earlier than white and Asian women (51, 52). Moreover, birth order is another potential confounding factor in the relationship between sociodemographic characteristics and MAR because individuals who delay childbearing often attempt to have their first child, and MAR is primarily used by those seeking to conceive their first child (14, 15).

The results of the multivariate analyses indicate that even after controlling for these factors associated with need for treatment, racial and educational gaps in MAR births persist. In particular, compared with white, NH fathers, black, NH fathers were associated with a decreased probability of having a MAR-conceived child, at −1.07 (95% CI, −1.11 to −1.04), net of other covariates. Statistically significant differences were also observed in the probability of a MAR-conceived birth on the basis of educational attainment, with fathers who had more than a 4-year degree being 2.04 percentage points (95% CI, 1.97–2.12) more likely to have a MAR-conceived birth tan fathers with less than a high school degree.

The results of the dyadic analyses suggest that the accumulation of educational advantage through forming a partnership with an individual with high educational attainment exacerbates the unequal access to MAR. This implies that partnering with an individual of similar or higher socioeconomic status further increases the likelihood of having a MAR-conceived birth beyond what would be observed by considering only individual-level characteristics. The results of the goodness-of-fit tests affirm the significance of considering the combined effect of both partners’ race and education when examining the impact of these variables on the probability of a birth being conceived through MAR.

The disparities in birth patterns by education may be explained by the greater ability of highly educated couples to pay for treatment. However, the persistence of racial gaps suggests that other factors are also at play. As our results show, race has an individual effect on the probability of having a MAR birth, with black, NH mothers and fathers having a significantly lower probability of conceiving using MAR than white, NH parents. Nevertheless, partnering with someone of a different race does not substantially change this probability (i.e., there is not a significant interactive effect between parental race characteristics).

The study by Greil et al. (42) found that black and Hispanic women with infertility were less likely to receive reproductive treatment than white and Asian women, even after taking into consideration factors such as income, education, and private insurance. Consistent with this study, our descriptive analysis suggests that partnering with someone who is not white or Asian reduces the likelihood of having a MAR-conceived child. However, results from the multivariate analysis indicate that the relationship between race and the incidence of MAR-conceived births is more complex than a simple binary classification. Because our study used birth records, it is worth noting that the observed disparities between parents of children conceived through MAR and those conceived naturally may be attributed to differences in the treatment success rates. For instance, the ART success rates have been shown to vary by race, with black and Hispanic women having lower success rates than white and Asian women (53, 54, 55). Further research is needed to gain a more comprehensive understanding of the intricate interplay between race and other contributing factors to the incidence of MAR-conceived children.

Other factors may contribute to the differences in the use of MAR, such as the profession of the parent, which could serve as a proxy for both wealth and ability to take time off for infertility treatments. Furthermore, a parent’s profession may be associated with the dependent variable used, such as education and race, potentially introducing a spurious association. Further research should explore these mechanisms and develop a testable theoretical framework for understanding how such disparities emerge and persist.

### Strengths and Limitations

This study has several strengths. First, the utilization of national-level data allowed for a comprehensive examination of the sociodemographic characteristics of parents involved in MAR births in the United States. Second, confounding variables, such as maternal age and birth order, were controlled for. In particular, the control for birth order is a unique feature of this study. Third, the dyadic focus of the study with the inclusion of men provided a clearer understanding of the relationship between MAR births and parental sociodemographic characteristics (17, 18, 19, 20, 21, 22, 23).

It is a limitation of this study that the observed disparities between parents of children conceived through MAR and those conceived naturally may not solely reflect differences in access to treatments. For instance, men and couples from lower socioeconomic backgrounds may have lower success rates when using ART or be less likely to seek help (55, 56). In addition, this study focused on the United States, and the findings may not be easily generalizable to other settings. Although data missingness may have biased our estimates, sensitivity analyses showed that the impact on our results was negligible. Finally, the NVSS data are known to underreport MAR births, which may lead to an underestimation of the contribution of MAR to total births. Moreover, it is not known whether this underreporting is patterned by sociodemographic characteristics or features relevant to this study (16, 57, 58).

## Conclusion

In conclusion, the results showed significant inequalities in the distribution of MAR births, with older, higher educated, and white, NH fathers being more likely to have a child conceived using MAR. This study controlled for maternal age and birth order, suggesting that socioeconomic and racial disparities stem from a combination of financial, behavioral, and cultural factors, emphasizing the complexity of access to MAR. The findings suggest that children born through MAR are raised in environments of relative advantage, which may impact their future health and development. This information is crucial for clinicians because it highlights how potential health and developmental disparities among children born through ART may be mediated by the social context in which they are raised. Additionally, the persistent socioeconomic inequalities in the ability to achieve a birth through MAR reveal a clear case of reproductive inequity, with significant policy implications.

## References

[1] Zegers-Hochschild F., Adamson G.D., Dyer S., Racowsky C., De Mouzon J., Sokol R. (2017). The international glossary on infertility and fertility care, 2017. Hum Rep.

[2] Stephen E.H., Chandra A., King R.B. (2016). Supply of and demand for assisted reproductive technologies in the United States: clinic- and population-based data, 1995-2010. Fertil Steril.

[3] Sunderam S., Zhang Y., Jewett A., Kissin D.M. State-specific assisted reproductive technology surveillance, United States: 2019 data brief. https://www.cdc.gov/art/state-specific-surveillance/2019/pdf/state-specific-art-surveillance-u.s.-2019-data-brief-h.pdf.

[4] De Geyter C., Wyns C., Calhaz-Jorge C., Mouzon J de, Ferraretti A.P., Kupka M. (2020). 20 years of the European IVF-Monitoring Consortium registry: what have we learned? A comparison with registries from two other regions. Hum Rep.

[5] Leridon H., Slama R. (2008). The impact of a decline in fecundity and of pregnancy postponement on final number of children and demand for assisted reproduction technology. Hum Rep.

[6] Leridon H., Shapiro D. (2017). Biological effects of first birth postponement and assisted reproductive technology on completed fertility. Population.

[7] Goldstein J.R., Sobotka T., Jasilioniene A. (2009). The end of “lowest-low” fertility?. Popul Dev Rev.

[8] Sobotka T., Zeman K., Lesthaeghe R., Frejka T. (2011). Postponement and recuperation in cohort fertility: new analytical and projection methods and their application. European Demographic Research Papers.

[9] É Beaujouan, Sobotka T. (2019). Late childbearing continues to increase in developed countries. Population Societies.

[10] Johnson-Hanks J.A., Bachrach C.A., Morgan S.P., Kohler H.P., Johnson-Hanks J.A., Bachrach C.A., Morgan S.P., Kohler H.-P. (2011). Understanding family change and variation: toward a theory of conjunctural action.

[11] Raymer J., Guan Q., Norman R.J., Ledger W., Chambers G.M. (2020). Projecting future utilization of medically assisted fertility treatments. Popul Stud (Camb).

[12] Lazzari E., Potančoková M., Sobotka T., Gray E., Chambers G.M. (2023). Projecting the contribution of assisted reproductive technology to completed cohort fertility. Popul Res Policy Rev.

[13] Tierney K. (2022). The future of assisted reproductive technology live births in the United States. Popul Res Policy Rev.

[14] Goisis A., Håberg S.E., Hanevik H.I., Magnus M.C., Kravdal Ø. (2020). The demographics of assisted reproductive technology births in a Nordic country. Hum Rep.

[15] Lazzari E., Gray E., Chambers G.M. (2021). The contribution of assisted reproductive technology to fertility rates and parity transition: an analysis of Australian data. Demogr Res.

[16] Tierney K., Cai Y. (2019). Assisted reproductive technology use in the United States: a population assessment. Fertil Steril.

[17] Almeling R., Waggoner M.R. (2013). More and less than equal: how men factor in the reproductive equation. Gend Soc.

[18] Saewyc E.M. (2012). What about the boys? The importance of including boys and young men in sexual and reproductive health research. J Adolesc Health.

[19] Almeling R. (2015). Reproduction. Annu Rev Sociol.

[20] Barratt C.L.R., De Jonge C.J., Sharpe R.M. (2018). ‘Man Up’: the importance and strategy for placing male reproductive health centre stage in the political and research agenda. Hum Rep.

[21] Johnson K.M., Greil A.L., Shreffler K.M., McQuillan J. (2018). Fertility and infertility: Toward an integrative research agenda. Popul Res Policy Rev.

[22] Ravitsky V., Kimmins S. (2019). The forgotten men: rising rates of male infertility urgently require new approaches for its prevention, diagnosis and treatment. Biol Reprod.

[23] Calvert J.K., Fendereski K., Ghaed M., Bearelly P., Patel D.P., Hotaling J.M. (2022). The male infertility evaluation still matters in the era of high efficacy assisted reproductive technology. Fertil Steril.

[24] Choufani S., Turinsky A.L., Melamed N., Greenblatt E., Brudno M., Bérard A. (2019). Impact of assisted reproduction, infertility, sex and paternal factors on the placental DNA methylome. Hum Mol Genet.

[25] Halvaei I., Litzky J., Esfandiari N. (2020). Advanced paternal age: effects on sperm parameters, assisted reproduction outcomes and offspring health. Reprod Biol Endocrinol.

[26] Kumar N., Singh A.K. (2015). Trends of male factor infertility, an important cause of infertility: a review of literature. J Hum Reprod Sci.

[27] Hotaling J.M., Davenport M.T., Eisenberg M.L., VanDenEeden S.K., Walsh T.J. (2012). Men who seek infertility care may not represent the general U.S. population: data from the National Survey of Family Growth. Urology.

[28] Persily J., Stair S., Najari B.B. (2020). Access to infertility services: characterizing potentially infertile men in the United States with the use of the National Survey for Family Growth. Fertil Steril.

[29] Datta J., Palmer M.J., Tanton C., Gibson L.J., Jones K.G., Macdowall W. (2016). Prevalence of infertility and help seeking among 15 000 women and men. Hum Reprod.

[30] Bratsberg B., Rogeberg O., Skirbekk V. (2020). Fathers of children conceived using ART have higher cognitive ability scores than fathers of naturally conceived children. Hum Reprod.

[31] Choi S.K.Y., Venetis C., Ledger W., Havard A., Harris K., Norman R.J. (2022). Population-wide contribution of medically assisted reproductive technologies to overall births in Australia: temporal trends and parental characteristics. Hum Reprod.

[32] Benyamini Y., Gozlan M., Kokia E. (2009). Women’s and men’s perceptions of infertility and their associations with psychological adjustment: a dyadic approach. Br J Health Psychol.

[33] Johnson K.M., Johnson D.R. (2009). Partnered decisions? U.S. couples and medical help-seeking for infertility. Fam Relat.

[34] Hammarberg K., Baker H.W., Fisher J.R.W. (2010). Men’s experiences of infertility and infertility treatment 5 years after diagnosis of male factor infertility: a retrospective cohort study. Hum Reprod.

[35] Martins M.V., Peterson B.D., Almeida V., Mesquita-Guimarães J., Costa M.E. (2014). Dyadic dynamics of perceived social support in couples facing infertility. Hum Reprod.

[36] Taylor L.C. (2018). The experience of infertility among African American couples. J Afr Am St.

[37] Lazzari E., Gray E., Baffour B. (2022). A dyadic approach to the study of perceived subfecundity and contraceptive use. Demogr Res.

[38] Stern J.E., Luke B., Hornstein M.D., Cabral H., Gopal D., Diop H. (2014). The effect of father’s age in fertile, subfertile, and assisted reproductive technology pregnancies: a population based cohort study. J Assist Reprod Genet.

[39] Pelikh A., Smith K.R., Myrskylä M., Goisis A. (2022). Medically assisted reproduction treatment types and birth outcomes: a between-family and within-family analysis. Obstet Gynecol.

[40] Inhorn M.C. (2020). Where has the quest for conception taken us? Lessons from anthropology and sociology. Reprod Biomed Soc Online.

[41] Fujimoto V.Y., Luke B., Brown M.B., Jain T., Armstrong A., Grainger D.A. (2010). Racial and ethnic disparities in assisted reproductive technology outcomes in the United States. Fertil Steril.

[42] Greil A.L., McQuillan J., Shreffler K.M., Johnson K.M., Slauson-Blevins K.S. (2011). Race-ethnicity and medical services for infertility: stratified reproduction in a population-based sample of U.S. women. J Health Soc Behav.

[43] Chin H.B., Howards P.P., Kramer M.R., Mertens A.C., Spencer J.B. (2015). Racial disparities in seeking care for help getting pregnant. Paediatr Perinat Epidemiol.

[44] Peck J.D., Janitz A., Craig L.B. (2016). Ethnic and racial differences in the prevalence of infertility: national survey of family growth (NSFG). Fertil Steril.

[45] Shirazi T.N., Rosinger A.Y. (2021). Reproductive health disparities in the USA: self-reported race/ethnicity predicts age of menarche and live birth ratios, but not infertility. J Racial Ethn Health Disparities.

[46] Wooldridge J.M. (2012).

[47] White I.R., Royston P., Wood A.M. (2011). Multiple imputation using chained equations: Issues and guidance for practice. Stat Med.

[48] Shapiro A.J., Darmon S.K., Barad D.H., Albertini D.F., Gleicher N., Kushnir V.A. (2017). Effect of race and ethnicity on utilization and outcomes of assisted reproductive technology in the USA. Reprod Biol Endocrinol.

[49] Guzzo K.B., Hayford S.R. (2020). Pathways to parenthood in social and family context: decade in review, 2020. J Marriage Fam.

[50] Martinez G., Daniels K. (2023). Fertility of men and women aged 15-49 in the United States: National Survey of Family Growth, 2015-2019. Natl Health Stat Report.

[51] Sweeney M.M., Raley R.K. (2014). Race, ethnicity, and the changing context of childbearing in the United States. Annu Rev Sociol.

[52] Martin J.A., Hamilton B.E., Osterman M.J.K., Driscoll A.K., Drake Patrick (2018). Births: final data for 2017. Natl Vital Stat Rep.

[53] Seifer D.B., Simsek B., Wantman E., Kotlyar A.M. (2020). Status of racial disparities between black and white women undergoing assisted reproductive technology in the US. Reprod Biol Endocrinol.

[54] Wellons M.F., Fujimoto V.Y., Baker V.L., Barrington D.S., Broomfield D., Catherino W.H. (2012). Race matters: a systematic review of racial/ethnic disparity in Society for Assisted Reproductive Technology reported outcomes. Fertil Steril.

[55] Quinn M., Fujimoto V. (2016). Racial and ethnic disparities in assisted reproductive technology access and outcomes. Fertil Steril.

[56] Anderson J.E., Farr S.L., Jamieson D.J., Warner L., Macaluso M. (2009). Infertility services reported by men in the United States: national survey data. Fertil Steril.

[57] Thoma M.E., Boulet S., Martin J.A., Kissin D. (2014). Births resulting from assisted reproductive technology: comparing birth certificate and National ART Surveillance System Data, 2011. Natl Vital Stat Rep.

[58] Moaddab A., Bateni Z.H., Dildy G.A., Clark S.L. (2016). Poor compliance and lack of improvement in birth certificate reporting of assisted reproductive technology pregnancies in the United States. Am J of Obstet Gynecol.

