# Supplementary material for: Parental sociodemographics of medically assisted reproduction births in the United States: a dyadic population-level study
Source: F S Rep. 2023 Jun 1;4(3):292–9. doi: 10.1016/j.xfre.2023.05.008 (PMC7615071; doi:10.1016/j.xfre.2023.05.008)
Supplement: Supplementary Tables [file mmc1.docx]

**Table S1.** Linear probability models for a MAR-conceived birth with interaction terms (2019 births).

|  | Coefficient | 95% CI |
| --- | --- | --- |
| **Father's education** |  |  |
| More than 4-years degree | 0.81 | (0.48; 1.14) |
| 4-years degree | -0.01 | (-0.10; 0.08) |
| some college | -0.07 | (-0.12; -0.02) |
| High school or less. (Ref.) | 0.00 |  |
| **Mother's education** |  |  |
| More than 4-years degree | 0.26 | (0.05; 0.46) |
| 4-years degree | -0.48 | (-0.54; -0.43) |
| some college | -0.32 | (-0.35; -0.28) |
| High school or less. (Ref.) |  |  |
| **Father's race** |  |  |
| White, NH (Ref.) | 0.00 |  |
| Black, NH | -0.84 | (-0.94; -0.75) |
| Asian, NH | 0.82 | (-0.66; -0.49) |
| Hispanic | -0.57 | (0.47; 1.17) |
| Other, NH | -0.43 | (-0.56; -0.30) |
| **Mother's race** |  |  |
| White, NH (Ref.) |  |  |
| Black, NH | -0.31 | (-0.53; -0.09) |
| Asian, NH | 0.33 | (-0.09; -0.58) |
| Hispanic | -0.58 | (-0.68; -0.48) |
| Other, NH | -0.30 | (-0.45; -0.14) |
| **Father's education X Mother's education** |  |  |
| Both high school or less (Ref.) |  |  |
| Both some college | 0.18 | (0.11; 0.26) |
| Some college X 4-years degree | 0.39 | (0.29; 0.49) |
| Some college X More than 4-years degree | 0.60 | (0.32; 0.89) |
| 4-years degree X some college | 0.49 | (0.37; 0.62) |
| Both 4-years degree | 0.90 | (0.78; 1.01) |
| 4-years degree X More than 4-Years degree | 1.32 | (1.08; 1.57) |
| More than 4-years degree X some college | 0.88 | (0.43; 1.32) |
| More than 4-years degree X 4-years degree | 1.13 | (0.77; 1.48) |
| Both more than 4-years degree | 1.36 | (0.95; 1.76) |
| **Father's race X Mother's race** |  |  |
| Both White (Ref.) |  |  |
| Both Black, NH | -0.03 | (-0.27;0.21) |
| Black, NH X Hispanic | 0.43 | (0.27; 0.58) |
| Black, NH X Asian | -0.53 | (-1.09; 0.03) |
| Black, NH X Other, NH | 0.47 | (0.25; 0.68) |
| Hispanic X Black, NH | 0.07 | (-0.22; 0.35) |
| Both Hispanic | -0.02 | (-0.15; 0.11) |
| Hispanic X Asian, NH | -1.30 | (-1.70; -0.09) |
| Hispanic X Other, NH | 0.22 | (-0.02; 0.45) |
| Asian, NH X Black, NH | -1.09 | (-2.11; -0.08) |
| Asian, NH X Hispanic | -0.51 | (-0.64; -0.38) |
| Both Asian, NH | -1.16 | (-1.68; -0.64) |
| Asian, NH X Other | -0.89 | (-1.63; -0.14) |
| Other X Black, NH | -0.18 | (-0.50; 0.14) |
| Other X Hispanic, NH | -0.06 | (-0.27; 0.14) |
| Other X Asian, NH | -0.99 | (-1.53; -0.46) |
| Other X Other | 0.18 | (-0.05; 0.41) |
| **Controls** |  |  |
| **Maternal age** | 0.36 | (0.36; 0.37) |
| **Birth order** |  |  |
| 1 (Ref.) |  |  |
| 2 | -1.42 | (-1.47; -1.38) |
| 3 | -2.81 | (-2.86; -2.77) |
| 4 and above | -3.56 | (-3.62; -3.50) |
| **AIC** | -3347406 |  |
| **BIC** | -3346835 |  |
| **N** | 3.214,645 |  |

Note: NH=non-Hispanic. Coefficients show the percentage point change in the probability of the birth being conceived using MAR. Results are obtained by fitting linear probability models. Source: Authors’ calculations based on the National Vital Statistics System (NVSS).

**Table S2**. Linear probability models for MAR-conceived births (2009-2019 births).

|  | Model 1 = Paternal sociodemographics | | Model 2 = Paternal and maternal sociodemographics | |
| --- | --- | --- | --- | --- |
|  | Coefficient | 95% CI | Coefficient | 95% CI |
| **Father's race** |  |  |  |  |
| White, NH (Ref.) | 0.00 |  | 0.00 |  |
| Black, NH | -0.90 | (-0.91; -0.89) | -0.64 | (-0.66; -0.62) |
| Hispanic | -0.86 | (-0.87; -0.86) | -0.51 | (-0.53; -0.49) |
| Asian, NH | -0.96 | (-0.98; -0.94) | -0.49 | (-0.54; -0.45) |
| Other, NH | -0.52 | (-0.55; -0.50) | -0.40 | (-0.43; -0.37) |
| **Mother's race** |  |  |  |  |
| White, NH (Ref.) |  |  | 0.00 |  |
| Black, NH |  |  | -0.33 | (-0.35; -0.31) |
| Hispanic |  |  | -0.41 | (-0.43; -0.39) |
| Asian, NH |  |  | -0.11 | (-0.14; -0.08) |
| Other, NH |  |  | 0.00 | (0.00; 0.00) |
| **Father's education** |  |  |  |  |
| More than 4-years degree | 1.88 | (1.85; 1.90) | 1.46 | (1.44; 1.49) |
| 4-years degree | 0.73 | (0.72; 0.75) | 0.60 | (0.59; 0.62) |
| some college | 0.06 | (0.05; 0.07) | 0.09 | (0.07; 0.10) |
| HS or less. (Ref.) | 0.00 |  | 0.00 |  |
| **Mother's education** |  |  |  |  |
| More than 4-years degree |  |  | 0.97 | (0.95; 0.99) |
| 4-years degree |  |  | 0.00 | (-0.01; 0.02) |
| some college |  |  | -0.19 | (-0.20; -0.18) |
| HS or less. (Ref.) |  |  | 0.00 |  |
| **Maternal age** | 0.29 | (0.29; 0.29) | 0.28 | (0.28; 0.29) |
| **Birth order** |  |  |  |  |
| 1 (Ref.) | 0.00 |  |  |  |
| 2 | -1.46 | (-1.48; -1.45) | -1.44 | (-1.45; -1.43) |
| 3 | -2.41 | (-2.43; -2.40) | -2.36 | (-2.37; 2.34) |
| 4+ | -3.06 | (-3.07; -3.04) | 2.97 | (-2.98; -2.95) |
| **Year of birth** |  |  |  |  |
| 2009 (Ref.) |  |  |  |  |
| 2010 | 0.12 | (0.10; 0.14) | 0.12 | (0.09;0.14) |
| 2011 | 0.15 | (0.13;0.17) | 0.15 | (0.12;0.17) |
| 2012 | 0.16 | (0.14;0.18) | 0.16 | (0.14;0.18) |
| 2013 | 0.15 | (0.13;0.17) | 0.15 | (0.13;0.17) |
| 2014 | 0.14 | (0.12;0.16) | 0.11 | (0.09;0.13) |
| 2015 | 0.14 | (0.14;0.17) | 0.13 | (0.11;0.15) |
| 2016 | 0.26 | (0.24;0.28) | 0.23 | (-0.21;0.25) |
| 2017 | 0.36 | (0.34;0.38) | 0.33 | (0.31;0.35) |
| 2018 | 0.38 | (0.36;0.40) | 0.35 | (0.33;0.37) |
| 2019 | 0.44 | (0.41;0.46) | 0.40 | (0.38;0.43) |
| **AIC** | -41400000 |  | -40600000 |  |
| **BIC** | -41400000 |  | -40600000 |  |
| **N** | 33,442,463 |  | 33,442,463 |  |

Note: NH=non-Hispanic. Coefficients show the percentage point change in the probability of the birth being conceived using MAR. Results are obtained by fitting linear probability models. Source: Authors’ calculations based on the National Vital Statistics System (NVSS).
